# Hypoxia/reoxygenation-experienced cancer cell migration and metastasis are regulated by Rap1- and Rac1-GTPase activation *via* the expression of thymosin beta-4

**DOI:** 10.18632/oncotarget.3218

**Published:** 2015-03-23

**Authors:** Jae-Wook Lee, Yun-Kyoung Ryu, Young-Hoon Ji, Joo Hyun Kang, Eun-Yi Moon

**Affiliations:** ^1^ Department of Bioscience and Biotechnology, Sejong University, Seoul 143–747, Korea; ^2^ Research Center for Radiotherapy, Korea Institute of Radiological and Medical Science, Seoul 139–709, Korea; ^3^ Molecular Imaging Research Center, Korea Institute of Radiological and Medical Science, Seoul 139–709, Korea

**Keywords:** thymosin beta-4, Rac1-GTPase, Rap1-GTPase, cancer cell migration, tumor metastasis

## Abstract

Signaling by small guanosine triphosphatases (GTPase), Rap1/Rac1, is one of the major pathways controlling cancer cell migration and tumor metastasis. Thymosin beta-4 (Tβ4), an actin-sequestering protein, has been shown to increase migration of cancer cells. Episodes of hypoxia and re-oxygenation (H/R) are an important phenomenon in tumor microenvironment (TME).

We investigated whether Tβ4 could play as an intermediary to crosstalk between Rac1- and Rap1- GTPase activation under hypoxia/reoxygenation (H/R) conditions. Inhibition of Tβ4 expression using transcription activator-like effector nucleases (TALEN) significantly decreased lung metastasis of B16F10 cells. Rac1 and Rap1 activity, as well as cancer cell migration, increased following induction of Tβ4 expression in normoxia- or H/R-experienced cells, but were barely detectable in Tβ4-depleted cells. Rap1-regulated Rac1 activity was decreased by a dominant negative Rap1 (Rap1N17), and increased by 8-(4-chloro-phenylthio)-2′-O-methyladenosine-3′,5′-cyclic monophosphate (CPT), a Rap1 activator. In contrast, a Rac1-specific inhibitor, NSC23766, and dominant negative Rac1 (Rac1N17) enhanced Tβ4 expression and aberrant Rap1 activity. While NSC23766 and Rac1N17 incompletely inhibited tumor metastasis *in vivo*, and H/R-experienced cancer cell migration *in vitro*, more efficient attenuation of cancer cell migration was accomplished by simultaneous inactivation of Rap1 and Rac1 with Rap1N17 and Rac1N17, respectively.

These data suggest that a combination therapy targeting both Rap1 and Rac1 activity may be an effective method of inhibiting tumor metastasis.

## INTRODUCTION

In the solid tumor microenvironment (TME), the oxygen supply to tumor cells is often diminished to ~10 mmHg (1.3% O_2_), compared with 40–60 mmHg (5.3–7.9% O_2_) in healthy cells [1, 2], due to the rapid growth of tumor cells, which outpaces the surrounding endothelial cells necessary to support blood vessel growth [3]. This decrease in oxygen tension leads to widespread hypoxia in solid tumors, and presents significant obstacles to cancer therapy. Hypoxia decreases the efficacy of cancer therapeutics [4], results in increased resistance to most anticancer drugs, and accelerates the rate of malignant progression and metastasis [5]. Episodes of hypoxia and re-oxygenation (H/R) are an important phenomenon in the TME. Induction of vascular endothelial growth factor (VEGF) expression in the hypoxic TME leads to increase in angiogenesis and re-oxygenation of the tumor [6]. Cytotoxic therapies, such as radiotherapy, are also responsible for re-oxygenation of hypoxic tumors through the killing oxygenated cells [7]. Repeated episodes of H/R greatly increase the metastatic potential of cancer cells [8, 9]. As tumor metastasis is responsible for over 90% of cancer deaths [6], understanding the mechanisms underlying tumor metastasis is of significant concern.

Upon migration to the parenchyma of the distal organs, tumor cells establish local microenvironments that facilitate their survival and proliferation [10]. Significant transitions occur during the course of cancer cell migration and adhesion, involving a wide variety of structural proteins, and a reorganization of the actin cytoskeleton [11, 12]. This cytoskeletal reorganization is dependent on small GTPases, including Rac1, Cdc42, and Rap1 [13–15], with significant crosstalk among proteins.

Rap1 is a member of the Ras family GTPases with ~50% homology to Ras [16, 17]. Rac1 is a member of the Rho family small GTPases (Rho/Rac/Cdc42), which are thought to be involved in the regulation of actin dynamics [12, 15, 18]. Following activation, Rac1 binds to the PAK1 binding domain (PBD) in P21-activated kinase 1 (PAK1) [14, 19, 20], leading to the formation of lamellipodia at the leading edge [15, 19]. Rap1 activation subsequently induces the accumulation of Rac1 [21]. E3B1, a regulator of Rac, potentiates EGF-induced activation of Rap1 [22], which in turn promotes cell spreading by targeting a specific subset of Rac guanine nucleotide exchange factors (GEF) to sites of cell–matrix contact [23]. Rac can be activated by cAMP/Epac1/Rap1 in the secretory pathway [24], but has been shown to be suppressed by these same molecules in epithelial cells [25], indicative of a complex regulatory environment, which is likely influenced by many factors. However, despite this abundant evidence of crosstalk between Rac1 and Rap1, little is known about the mechanisms regulating these interactions. We are therefore interested in identifying molecules capable of regulating this crosstalk, sequentially, synergistically, or antagonistically.

Rapid cycles of actin assembly and disassembly require a number of actin binding proteins, including the monomeric G-actin-sequestering β-thymosins [26, 27], the actin-binding competitor profilin [28], and the F-actin-depolymerising cofilin [29]. Among the β-thymosins, thymosin beta-4 (Tβ4) is one of the most abundant member of the highly conserved polar 5-kDa peptides [30]. Originally isolated from the thymus, this small, naturally occurring 43 amino acid peptide has been shown to be present in all cell types, with the exception of erythrocytes [31, 32].

Tβ4 protein has been implicated in a wide variety of cancers due to its role in cytoskeletal reorganization. Tβ4 proteins form 1:1 complexes with G-actin [27], and regulate a diverse array of cellular functions, including intracellular signal transduction and cytoskeleton structure [33, 34]. Expression of this protein has been directly associated with increased tumor growth and metastasis [35], through mechanisms including anti-apoptosis resistance, paclitaxel-resistance through ROS production, and HIF-1α stabilization through Erk activation [4, 36]. In addition, Tβ4 is a hypoxia-responsive regulator, which controls cancer cell migration in angiogenesis and tumor metastasis [35, 37]. Tβ4 triggers epithelial-mesenchymal transition by up-regulating integrin-linked kinase [38], and plays a role in malignant progression and invasion in colon adenocarcinoma [39, 40]. In addition, Tβ4 in gastric cancer cells regulates Wnt signaling pathways [41]. However, little is known regarding the effects of Tβ4 on Rap1/Rac1 activation and Rap1- or Rac1-mediated cancer cell migration.

Here, we examined whether Tβ4 expression is associated with activation of Rac1 and Rap1, and the effect of this association on the crosstalk between Rac1 and Rap small GTPases in terms of cancer cell migration and tumor metastasis. As Rac1 and Rap1 regulate migration and metastasis in H/R-experienced cancer cells, we used HeLa cervical tumor cells and Tβ4-transgenic (Tg) mice to assess the effect of Tβ4 on Rac1- and Rap1-GTPase. We found that Rac1 and Rap1 activity was increased by Tβ4 expression both *in vitro* and *in vivo*. Rap1 activation was found to be enhanced following treatment with a Rac1-specific inhibitor, (NSC23766) or dominant negative Rac1 (Rac1N17), indicative of a Tβ4-mediated compensatory feedback mechanism. The greatest inhibition of cancer cell migration was seen following simultaneous inactivation of Rap1- and Rac1-GTPase.

## RESULTS

### Tβ4 expression was correlated with the activation of Rap1- and Rac1-GTPases

**Figure 1:**
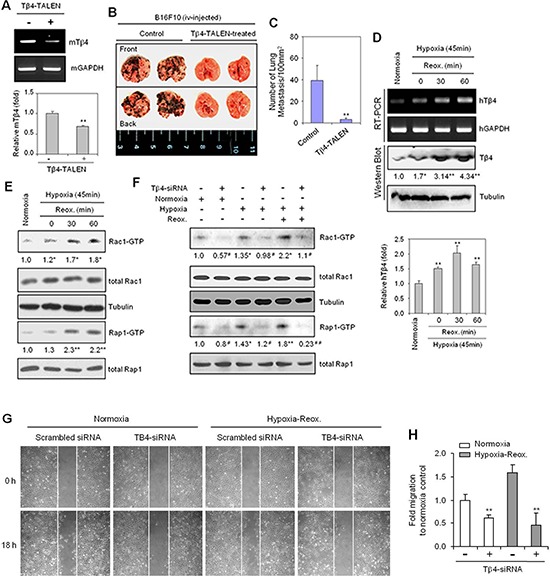
Thymosin beta 4 (Tβ4) expression is correlated with Rac1- and Rap1-GTPase activation, lung metastasis, and cell migration in hypoxia-reoxygenation (H/R)-experienced cancer cells **(A–C)** B16F10 cells were cultivated *in vitro* in log phase and transfected with Tβ4-TALEN. Tβ4 expression in Tβ4-TALEN-treated B16F10 cells was detected by RT-PCR (A, top) or realtime PCR (A, bottom). Five 7-week-old C57BL/6 wild-type mice were injected with 2 × 10^5^ B16F10 control or Tβ4-TALEN-treated cells *via* tail-vein injection. All mice were sacrificed 14 d after tumor injection. Lung metastasis was shown in the photograph (B). The degree of lung metastasis was assessed by counting tumor colonies under a light dissection microscope (C). **(D and E)** HeLa cells were subjected to reoxygenation for 30 or 60 min following incubation in a hypoxia chamber for 45 min. RNA was isolated, and Tβ4 transcript levels were measured by RT-PCR (D, top, upper) or realtime PCR (D, bottom). Tβ4 protein levels were detected by western blotting (D, top, lower). Rac1 and Rap1 activities were examined using a GST-pulldown assay targeting the RBD domain, and visualized by western blotting (E). **(F)** HeLa cells were transfected with scrambled control siRNA or Tβ4-siRNA, respectively and incubated for 24 h prior to incubation under hypoxia (45 min) and reoxygenation (60 min) conditions. Rac1 and Rap1 activities were detected by GST-pulldown and western blotting. **(G and H)** HeLa cells transfected with scrambled control siRNA or Tβ4-siRNA were plated on 35-mm^2^ dishes and incubated under normoxic conditions for 24 h. A confluent monolayer of HeLa cells was then scratched with a sterile pipet tip, and incubated in normoxia or hypoxia for 45 min, followed by reoxygenation for 18 h. Migration of cells into the space left by the scratch was photographed using a phase-contrast microscope at 200× magnification (G). The empty area remaining at each time point was quantified using NIH image analysis software (version 1.62), and compared to that of the 0-h time point (H). Data shown are representative of three independent experiments (A–H). Data in bar graph are presented as means ± SD (A, C, D, and H). Band intensities were normalized relative to controls using NIH image analysis software (Image J, version 1.62). Fold changes relative to the control are indicated under each band (D–F). *p < 0.05; **p < 0.01 relative to the control (A–H).

To examine the possibility that Tβ4 interacts with Rap1, we performed a yeast two-hybrid screen using the RalGDS-Ras binding domain (RBD), which binds active Rap1-GTP, as a bait. The initial screen revealed 86 colonies that exhibited strong blue signals (Supplementary Figure 1), suggesting that Tβ4 may be a regulator of Rap1 GTPase activation. Furthermore, as Rap1 and Rac together regulate the secretion of sAPPalpha [24], we examined the effect of Tβ4 on lung metastasis *in vivo*. B16F10 cells were transfected with a transcription activator-like effector nuclease (TALEN) targeting Tβ4, effectively suppressing Tβ4 expression using RT-PCR (Figure 1A, top) and realtime PCR (Figure 1A, bottom). These cells were then injected into the tail vein of C57BL/6 mice. Tumor metastasis was found to be reduced in mice injected with the Tβ4-TALEN-transfected cells relative to control cells (Figure 1B and 1C), suggesting that tumor metastasis may be associated with Tβ4 signaling pathways.

Hypoxia and reoxygenation (H/R) is an important phenomenon in the tumor microenvironment, as they lead to drug resistance and an increase in cancer cell migration [31, 42]. We therefore examined whether H/R could enhance Tβ4 gene expression. Both Tβ4 gene expression using RT-PCR (Figure 1D, top, upper) or realtime PCR (Figure 1D, bottom) and protein abundance (Figure 1D, top, lower) were increased under conditions of hypoxia, as compared to normoxia; these effects were further amplified following H/R. As H/R has previously been shown to increase metastatic potential in tumors [6, 7], we examined HeLa cell migration under H/R conditions. HeLa cells were pre-incubated in a hypoxia chamber for 45 min, followed by a return to normoxic conditions. Cell migration was increased 1.7-fold under H/R conditions relative to normoxic conditions (Supplementary Figure 2A and 2B).

Given that Rac1 and Rap1 play an important role in cell migration [13, 43], we examined whether these proteins were activated in HeLa cells under hypoxia or H/R conditions. Rac1 and Rap1 activity increased in a time-dependent manner in response to hypoxic conditions (Supplementary Figure 2C), and following H/R, as compared to that in normoxia (Figure 1E). Confirmation of hypoxic conditions was determined based upon an increase in HIF-1α stabilization (Supplementary Figure 2C). These data suggest that Tβ4 expression could be associated with cancer cell migration and the activation of GTPase, Rac1 and Rap1, in H/R conditions.

To examine the relationship between Tβ4 expression and Rap1/Rac1 GTPase activation under H/R conditions, we transfected cells with Tβ4-siRNA to inhibit Tβ4 expression. Both Rac1 and Rap1 activity were decreased in Tβ4-siRNA-transfected cells under normoxic and H/R conditions (Figure 1F). In addition, cell migration was reduced in Tβ4-siRNA-transfected cells under normoxic or H/R conditions (Figure 1G). The percentage of inhibition was ~70% in cells subjected to H/R, compared to only ~30% in cells under normoxia (Figure 1H). Together, these data are consistent with a role for Tβ4-mediated activation in cancer cell migration *via* its regulation of Rap1 and Rac1 GTPase activation.

### The GTPase, Rac1 and Rap1 activity is dependent on Tβ4 expression

**Figure 2:**
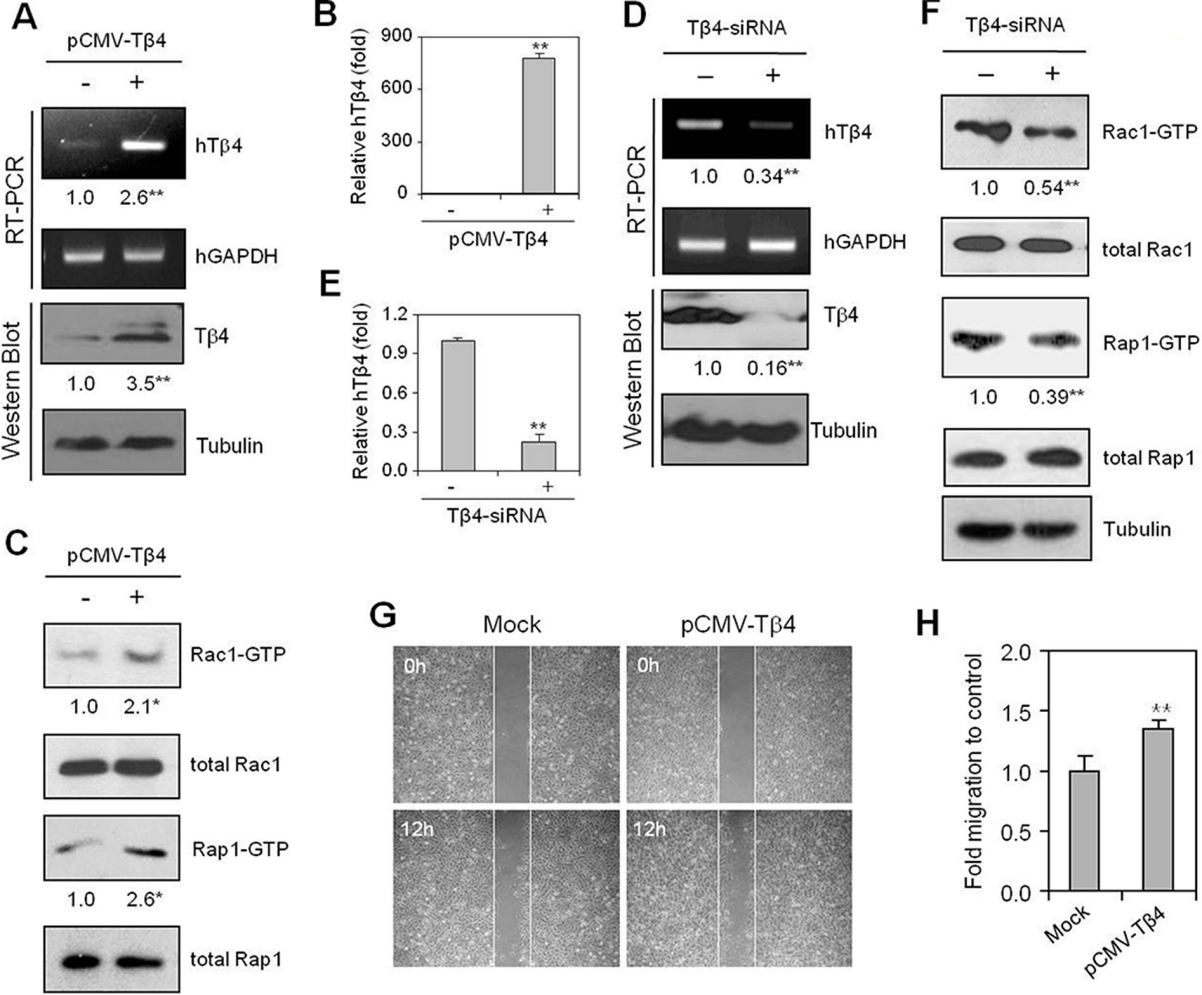
Cancer cell migration is dependent on Tβ4-mediated activation of Rap1- and Rac1-GTPases **(A–C)** HeLa cells were transfected with pCMV-Tβ4 plasmid for 24 h. Tβ4 transcript levels were measured by RT-PCR (A, upper) or realtime PCR (B). Tβ4 protein levels were determined by western blotting (A, lower). Rac1 and Rap1 activity were detected by GST-pulldown and western blotting, as described in the Materials and methods (C). **(D–F)** Cells were transfected with scrambled control siRNA or Tβ4-siRNA, and incubated for 24 h, after which RNA was isolated. Tβ4 transcript levels were measured by RT-PCR (D, upper) or realtime PCR (E). Tβ4 protein levels were detected by western blotting (D, lower). Rac1 and Rap1 activities were detected by GST-pulldown and western blotting (F). **(G and H)** HeLa cells were plated on 35-mm^2^ dishes and incubated under normoxic conditions for 24 h, and then transfected with either control vector (mock) or pCMV-Tβ4 plasmids for 18 h. A confluent monolayer of HeLa cells was then scratched with a sterile pipet tip, and incubated for 12 h under normoxic conditions. Migration of cells into the space left by the scratch was photographed using a phase-contrast microscope at 200× magnification (G). The empty area remaining at 12 h was quantified using NIH image analysis software (version 1.62), and compared to that of the 0-h time point (H) Data shown are representative of three independent experiments (A–H). Band intensities were normalized relative to controls using NIH image analysis software (Image J, version 1.62). Fold changes relative to the control are indicated under each band (A, C, D, and F). Data in bar graph are presented as means ± SD (B, E, and H). *p < 0.05; **p < 0.01 relative to the control (A–H).

To re-examine whether Rac1 and Rap1 activity was dependent on Tβ4 expression under normoxic conditions, we modified Tβ4 expression in HeLa cells using a pCMV-Tβ4 plasmid or Tβ4-siRNA. Overexpression of Tβ4 *via* the transfection of HeLa cells with pCMV-Tβ4 (Figure 2A and 2B) led to increased activity of both Rac1 and Rap1 relative to that of empty vector controls (Figure 2C). In contrast, when cells were treated with Tβ4-siRNA, Tβ4 expression was inhibited significantly (Figure 2D and 2E). The Tβ4 knockdown inhibited Rac1 and Rap1 activity (Figure 2F).

Cancer cell migration was significantly enhanced following transfection with a pCMV-Tβ4 plasmid under normoxic conditions (Figure 2G). The mobility of Tβ4-overexpressing cells was 30% higher than that of controls (Figure 2H), suggesting that cancer cell migration may be dependent on Tβ4-mediated activation of Rap1- and Rac1-GTPases.

### NSC23766, a Rac1 inhibitor, increases Tβ4-mediated Rap1 activity

**Figure 3:**
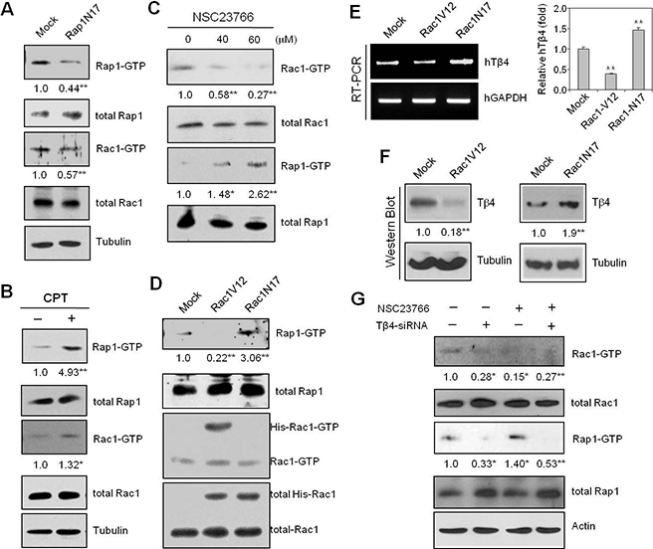
Rap1-GTPase is activated following inhibition of Rac1-GTPase with NSC23766 or Rac1N17 **(A)** HeLa cells were transfected with Rap1N17 or control plasmid (Mock) vectors for 24 h. Rac1 and Rap1 activity were detected by GST-pulldown and western blotting. **(B)** HeLa cells were treated with 50 μM CPT, and GST-pulldown was used to detect Rap1 and Rac1 activation. **(C)** HeLa cells were treated with a Rac1-GTPase inhibitor, NSC23766 (40 or 60 μM), or DMSO for 12 h. Rac1 and Rap1 activities were detected by GST-pulldown and western blotting. **(D–F)** HeLa cells were transfected with His-Rac1 V12, His-Rac1 N17, or pCDNA3.1 control (Mock) plasmid vectors for 24 h, and assayed for Rac1 and Rap1 activities by GST-pulldown and western blotting (D). Tβ4 transcript levels were measured by RT-PCR (E, left) or realtime PCR (E, right). Tβ4 protein levels were detected by western blotting (F). **(G)** Cells were transfected with siRNA-Tβ4 or scrambled control siRNA for 24 h, and incubated in the presence or absence of NSC23766; Rac1 and Rap1 activities were detected by GST-pulldown and western blotting. Data shown are representative of three independent experiments (A–G). Band intensities were normalized relative to controls using NIH image analysis software (Image J, version 1.62). Fold changes relative to the control are indicated under each band (A–D, F, and G). Data in bar graph are presented as means ± SD (E). *p < 0.05; **p < 0.01 relative to the control (A–G).

To confirm that Rap1 is associated with Rac1 activation and cell migration, we used dominant negative Rap1(Rap1N17) plasmids or 8-(4-Chlorophenylthio) adenosine 3′,5′-cyclic monophosphate (CPT), an Epac-specific cAMP analog, to modify Rap1. Both Rap1 and Rac1 activities were effectively inhibited following transfection with Rap1N17 plasmids (Figure 3A), but enhanced following treatment with CPT (Figure 3B). Alternatively, treatment with NSC23766, a Rac1 inhibitor, led to a decrease in Rac1 activity, but an increase in Rap1 activation (Figure 3C).

To confirm that Rac1 regulates Rap1 activation, we repeated the above experiments using constitutively active (Rac1V12) and dominant-negative Rac1 mutants (Rac1N17). Rac1V12 exhibited decreased Rap1 activity compared to Rac1N17, which showed higher overall Rap1 activity (Figure 3D). Similarly, Tβ4 transcript and protein levels were decreased by Rac1V12, but were increased by Rac1N17, which was measured by RT-PCR (Figure 3E, left), realtime PCR (Figure 3E, right), and western blotting (Figure 3F). Finally, a small amount of Rap1 activity was detected following incubation with Rac1 inhibitor, NSC23766, which was barely observed by the inhibition of Tβ4 expression with Tβ4-siRNA (Figure 3G). Taken together, these data suggest that Rac1 inhibition may be associated with an increase in Tβ4-mediated Rap1 activation.

### Rac1 inhibition decreases tumor metastasis *in vivo* but induces Tβ4 expression

**Figure 4:**
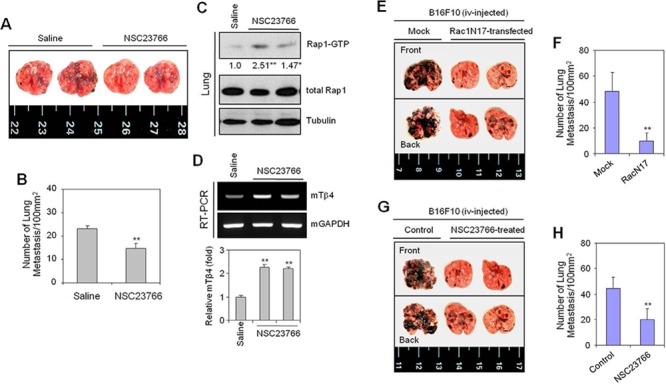
The Rac1-GTPase inhibitor, NSC23766, or Rac1N17 inhibited tumor metastasis *in vivo* **(A–D)** B16F10 cells were cultivated *in vitro* to log phase. Five wild-type C57BL/6 mice were inoculated with 2 × 10^5^ B16F10 cells by tail vein injection. NSC23766 was then injected intraperitoneally at a dose of 2.5 mg/kg every 12 h for 12 d. Mice were then sacrificed on day 19, and examined for lung metastasis (A). The number of lung metastases was assessed by counting tumor colonies under a light dissection microscope (B). Rap1 activity in NSC23766-treated and control B16F10 tumor-bearing mouse lung tissues was detected by GST-pulldown and western blotting. Band intensities were normalized relative to controls using NIH image analysis software (Image J, version 1.62). Fold increases relative to the control are indicated under each band (C). RNA was isolated from control and NSC23766-administered mouse lung tissues. Tβ4 transcript levels were measured by RT-PCR (D, top) or realtime PCR (D, bottom). Data in bar graph are presented as means ± SD (B and D). *p < 0.05; **p < 0.01 relative to saline control (B–D). **(E–H)** B16F10 cells were transfected with Rac1N17 (E and F), or treated with NSC23766 (G and H) for 24 h. Five wild-type C57BL/6 mice were inoculated with 2 × 10^5^ Rac1N17-transfected (E and F) or NSC23766-treated (G and H) B16F10 cells by tail-vein injection. Mice were then sacrificed on day 14, and examined for lung metastasis. (E and G). The number of lung metastases was assessed by counting tumor colonies under a light dissection microscope. Data in bar graph are presented as means ± SD (F and H). **p < 0.01 relative to mock-treated (F) or B16F10 cell-injected controls (H). Data shown are representative of three independent experiments (A-H).

Given that cell migration plays an important role in many physiological and pathological processes, including tumor metastasis [18], we examined the effect of Rac1 inhibition on cancer cell migration in vivo. Lung metastasis of B16F10 tumor cells was inhibited by the administration of NSC23766 (Figure 4A). The number of tumor colonies was significantly decreased in NSC23766-administered mice, compared to untreated controls, however a residual number of colonies remained (Figure 4B), indicative of compensatory activation of other proteins. Indeed, Rap1 activity was significantly increased by administration of NSC23766 (Figure 4C), as was Tβ4 gene expression in the lungs of NSC23766-administered mice using RT-PCR (Figure 4D, upper) and realtime PCR (Figure 4D, lower). Data demonstrate that Rac1 inhibition is not only effective in early time points *in vitro* (Figures 1 and 3) but also in later time points *in vivo*.

To confirm the effect of Rac1 activity on tumor metastasis *in vivo*, we examined lung metastasis in mice injected with Rac1N17-transfected or NSC23766-treated B16F10 cells. Lung metastasis was inhibited by ~80% in the group injected with Rac1N17-transfected B16F10 cells, relative to controls (Figure 4E and 4F), and by ~50% in the group injected with NSC23766-treated B16F10 cells (Figure 4G and 4H). Rac1 activity may therefore represent a novel target for the control of tumor metastasis *in vivo*.

Together, these data suggest that Rac1 inhibition alone is insufficient to control tumor metastasis. This incomplete inhibition of tumor metastasis following Rac1-inhibition may be the result of a compensatory activation of Tβ4-mediated Rap1, suggesting that both Rac1- and Rap1-GTPase activity may be regulated by Tβ4.

### Rac1 inhibition partially inhibits cancer cell migration under H/R conditions

**Figure 5:**
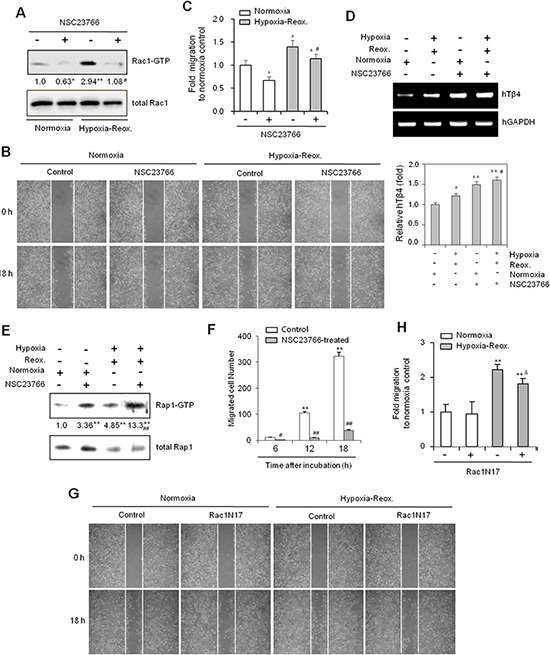
The Rac1-GTPase inhibitor NSC23766 inhibits cancer cell migration under hypoxia-reoxygenation conditions *in vitro* **(A–E)** HeLa cells were treated with NSC23766 (40 μM) or DMSO, and incubated under normoxic or hypoxic conditions for 45 min, followed by reoxygenation for 60 min. Rac1 activity was detected by GST-pulldown and western blotting (A). A confluent monolayer of HeLa cells was then scratched with a sterile pipet tip, and incubated for 18 h under normoxic conditions. Migration of cells into the space left by the scratch was photographed using a phase contrast microscope at 200× magnification (B). The empty area remaining at 18 h was quantified using NIH image analysis software (Image J, version 1.62), and compared to that of the 0-h time point (C). RNA was isolated from cells, and Tβ4 transcript levels measured by RT-PCR (D, top) or realtime PCR (D, bottom). Rap1 activity was detected by GST-pulldown and western blotting (E). Band intensities were normalized relative to controls using NIH image analysis software (Image J, version 1.62). Fold changes relative to the control are indicated under each band A, (E). Data in bar graph are presented as means ± SD (C and D). *p < 0.05; **p < 0.01 relative to NSC23766-untreated normoxia control. ^#^*p* < 0.05; ^##^*p* < 0.01 relative to NSC23766-untreated hypoxia-reoxygenation group (A–E). **(F)** NSC23766-treated HeLa cells were subjected to a cell migration assay using a Boyden chamber. Migrated cells were counted at 6, 12, and 18 h after incubation. Data are presented as means ± SD. **p < 0.01 vs. control at the 0-h time point. ^##^*p* < 0.01 relative to NSC23766-untreated group at each time point. **(G and H)** HeLa cells were then transfected with Rac1N17 or control plasmid vectors for 6 h. A confluent monolayer of HeLa cells was scratched with a sterile pipet tip and incubated for 45 min under normoxia or hypoxia, followed by reoxygenation for 18 h. Migration of cells into the space left by the scratch was photographed using a phase contrast microscope at 200× magnification (G) The empty area remaining at 18 h was quantified using NIH image analysis software (Image J, version 1.62), and compared to that of the 0-h time point. Data in bar graph are presented as means ± SD. **p < 0.01 relative to NSC23766-untreated normoxia control. ^&^*p* < 0.05 relative to Rac1N17-untreated hypoxia-reoxygenation group (H). Data shown are representative of three independent experiments (A–H).

To re-affirm the effect of Rac1 inhibition on cancer cell migration, H/R-experienced cells were incubated with NSC23766. Rac1 activity was increased under H/R conditions, but was effectively inhibited by NSC23766 (Figure 5A). Cancer cell migration was also decreased following NSC23766 treatment under both normoxic and H/R conditions (Figure 5B). NSC23766 inhibited cancer cell migration by ~30% under normoxic and ~20% under H/R conditions, relative to untreated controls (Figure 5C). However, the migration of H/R-experienced NSC23766-treated cells was comparable to that of untreated controls maintained under normoxic conditions. As before, this incomplete inhibition of caner cell migration may be the result of compensatory activation of other molecules associated with cancer cell migration.

Given that Rap1 activation is involved in tumor metastasis [13, 43] through the activation of Rac1 [23, 24], we next examined the effect of NSC23766 on Rap1 activation. Tβ4 gene expression (Figure 5D) and Rap1 activity (Figure 5E) were significantly increased following treatment with NSC23766 under both normoxic and H/R conditions. The inhibitory effect of NSC23766 on cell migration was confirmed using a Boyden chamber assay (Figure 5F). In addition, *in vitro* cell migration was inhibited ~20% following Rac1N17 transfection (Figure 5G and 5H). Together, these data confirmed that cancer cell migration in H/R-experienced cells could not be controlled by inhibition of Rac1 activity alone. This effect may be the result of Tβ4-mediated Rap1 activation by Rac1 inhibition. Such a hypothesis is consistent with the increases in Tβ4-mediated Rap1 activity in NSC23766-treated mice (Figure 4). Cancer cell migration in Rac1 inhibitor-treated H/R-experienced cells may therefore be maintained by a compensatory increase in Tβ4-mediated Rap1 activity.

### Cancer cell migration is efficiently attenuated by combined Rap1/Rac1 inhibition

**Figure 6:**
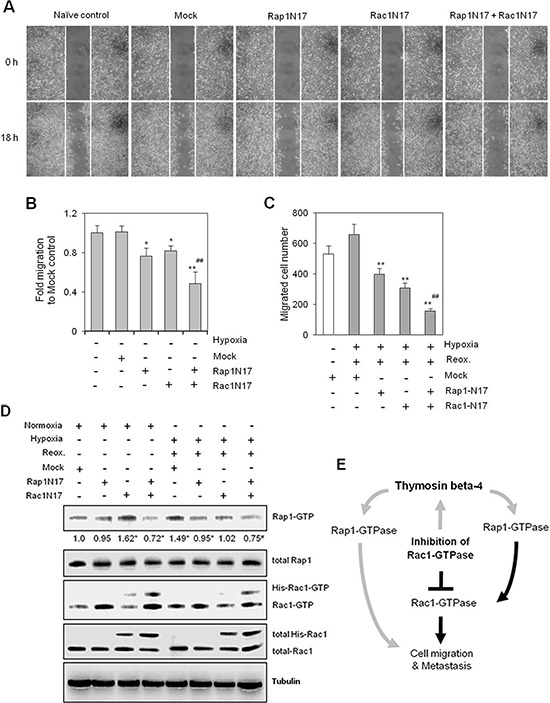
Simultaneous inhibition of Rac1- and Rap1-GTPases inhibited cancer cell migration more effectively than Rac1 or Rap1 alone **(A–D)** HeLa cells were plated onto 35-mm^2^ dishes, and incubated for 24 h under normoxic conditions. Cells were then transfected with Rac1-N17, Rap1-N17, or pCDNA3.1 control plasmid (Mock) vectors both alone and in combination, and then incubated for 6 h. A confluent monolayer of HeLa cells was then scratched with a sterile pipet tip, and incubated for 18 h under normoxic conditions. The migration of cells into the space left by the scratch was photographed using a phase-contrast microscope at 200× magnification (A). The empty area remaining at 18 h was quantified using NIH image analysis software (Image J, version 1.62), and compared to that of the 0-h time point (B). HeLa cells transfected with Rap1N17 or/and Rac1N17 were subjected to migration assays using a Boyden chamber. Cells were incubated under normoxia or hypoxia for 45 min, followed by reoxygenation for 18 h. Migrated cells were counted 18 h after incubation (C). Rac1 and Rap1 activities were detected by GST-pulldown and western blotting. Band intensities were normalized relative to controls using NIH image analysis software (Image J, version 1.62). Fold changes relative to the control are indicated under each band (D). Data shown are representative of three independent experiments (A–D). Data in bar graph are presented as means ± SD (B and C). *p < 0.05; **p < 0.01 relative to mock-treated control group. ^#^*p* < 0.05; ^##^*p* < 0.01 relative to Rac1N17- or Rap1N17-treated normoxia (B) or hypoxia-reoxygenation (C and D) group. **(E)** Hypothetical model of Tβ4-mediated Rap1-GTPase signaling in tumor metastasis and cancer cell migration. Rap1 affects tumor metastasis and cancer cell migration directly or indirectly through Rac1 activation. When Rac1 activation is blocked by a Rac1-GTPase specific inhibitor, cancer cell motility, in terms of both metastasis and migration, is maintained by Rap1 activation as a result of Tβ4 expression. Gray lines represent novel mechanisms identified in this study.

To assess the effect of compensatory feedback activation of Rap1 on cancer cell migration, we treated cells with Rap1N17 and/or Rac1N17 to inhibit Rap1 and Rac1, respectively. Cell migration was inhibited in both Rap1N17- and Rac1N17-transfected cells, relative to controls. Synergistic effects were seen following co-transfection with both Rap1N17 and Rac1N17, with cell migration significantly lower than that of individual treatments alone (Figure 6A). Cell migration was inhibited by ~25% and 20% in Rap1N17- and Rac1N17-treated cells, respectively, compared to ~50% in Rap1N17 and Rac1N17 co-transfected cells (Figure 6B). Additional inhibitory effects of Rap1N17 or/and Rac1N17 were confirmed using a Boyden chamber under H/R conditions (Figure 6C). In addition, Rap1 and Rac1 activity were inhibited by Rap1N17 and Rac1N17, respectively, under normoxic and H/R conditions. Rap1 activity was increased in Rac1N17-treated cells under normoxic conditions (Figure 6D), which is consistent with the results shown in Figure 4, although the effects of these inhibitors on the individual signaling molecules driving these phenotypes is unknown. Taken together, the data presented here highlight the role of Tβ4-mediated Rap1 activation as an encouraging target for the prevention of cell migration in Rac1-inhibitor-treated cells by preventing compensatory activation of Rap1 (Figure 6E).

## DISCUSSION

The alteration of oxygen tension in solid tumors leads to anticancer drug resistance, accelerated malignant progression, and tumor metastasis [5]. The hypoxic tumor region is re-oxygenated by angiogenesis, and through the killing oxygenated cells by the treatment with anticancer therapeutics [6, 7, 37]. This cycle of hypoxia and re-oxygenation enhances cancer cell migration [14, 20] through its effects on a wide variety of proteins associated with the re-organization of the actin cytoskeleton [11, 12, 18, 33, 34]. Among the most important proteins in cytoskeletal rearrangements are the small GTPases, including Rac1, Cdc42, and Rap1 [13–15], which are bound to a series of actin-binding proteins, including monomeric G-actin-sequestering β-thymosins [26, 27]. Tβ4 regulates intracellular signal transduction and cytoskeleton structure [26]; however, little is known regarding its relationship to Rac1 and Rap1.

Here, we examined the effects of Tβ4 on the interaction between Rac1 and Rap1. We found that Tβ4 activated both Rap1 and Rac1 in tumor cells. This regulation was found to be important for tumor metastasis *in vivo* (Figure 1). Cancer cell migration was also dependent on Tβ4-induced activation of Rap1 and Rac1 (Figures 1 and 2). Rap1 was activated in response to Rac1-specific inhibition (Figure 3), leading to an incomplete inhibition of *in vivo* tumor metastasis and *in vitro* cancer cell migration (Figures 4 and 5). Accordingly, cancer cell migration was inhibited more efficiently by simultaneous inactivation of Rap1 and Rac1 with Rap1N17 and Rac1N17, respectively (Figure 6). This suggests that Tβ4 may act as a central mediator of Rap1 and Rac1 activation, and that together, Rap1 and Rac1 could be an efficient therapeutic target for the prevention of tumor metastasis and cancer cell migration.

Despite the apparent links between Tβ4 and Rap1, direct evidence of this interaction remains elusive. The data presented here showed that Rap1 activity was increased in Tβ4-overexpressing cells (Figure 2B). In contrast, Rap1 activity was decreased by the inhibition of Tβ4 expression with Tβ4-siRNA (Figure 2D). Moreover, an increase in Rap1 activity was correlated with the induction of Tβ4 in Rac1-inhibited cells (Figure 3C, 3D, 3E, and 3F), suggesting that Rap1 likely acts downstream of Tβ4.

Furthermore, Rap1 and Rac1 were suppressed by Tβ4-siRNA in both normoxia- and H/R-experienced cells (Figure 1F). Rac1 inhibitors, NSC23766 or Rac1N17, induce Rap1 and Tβ4 expression in both normoxia- and H/R-experienced cells (Figure 5). However, the effect of Tβ4-siRNA and Rac1 inhibitor was higher in H/R-experienced cells as compared to the cells in normoxia. Then, we focus on H/R-experienced cancer cell migration and metastasis, which are regulated by crosstalk between Rap1 and Rac1 *via* Tβ4 expression.

Because cancer cell migration remained higher than basal level, and Tβ4 was up-regulated in NSC23766- or Rac1N17-treated cells, other migration-associated molecules may also be controlled by Tβ4. It is thought that other migration-associated molecules play a role in cancer cell migration when Rac1 activity is inhibited. As Rap1 activation is involved in migration, invasion, and metastasis through the regulation of metalloproteinase and integrin secretions [13, 21, 43], we assume that Tβ4-mediated Rap1 activation in Rac1-inhibited cells acts as a compensatory mechanism in response to reduced motility. In other words, Tβ4 may act as a regulator of cancer cell migration and tumor metastasis by regulating the interaction between Rac1 and Rap1 activity.

Although the incidence of lung metastasis was reduced following treatment with a Rac1 inhibitor, lung metastatic lesions in NSC23766-treated mice could be related to an increase in Rap1 activity. Furthermore, we cannot rule out Rac1 participation in a negative feedback loop with Rap1 [21, 44]. Our data showed that Rac1V12 inhibited Tβ4 protein expression (Figure 3E), which could be interpreted as a negative feedback mechanism regulating Rap1 activity. It is also possible that migration-associated molecules other than Rap1 play a role in cancer cell migration upon inhibition of Rac1 activity, or vice versa (Figure 6D). Tβ4-mediated Wnt signaling pathways [41] could be also associated with Rap1 activation in Rac1-inhibited cancer cells.

Ras protein is involved in tumorigenesis and transduction of signals *via* Raf, phosphatidylinositol-3 kinase, and phospholipase C [45]. However, the Ras pathway *via* RalGDS has not been investigated thoroughly. Our yeast two-hybrid assay showed that Tβ4 bound the RalGDS domain (RBD), which in turn bound active Rap1-GTP. This could explain Tβ4-mediated Rap1 activation in NSC23766-treated cells and tissues, as follows: NSC23766-mediated induction of Tβ4 may activate GEFs that exchange GDP with GTP in Rap1. Active Rap1 could then bind to Tβ4 in actin cytoskeleton complex structures, indirectly stabilizing Rap1-GTP *via* the RBD domain during cancer cell migration. Another possible explanation for the incomplete attenuation of cancer cell migration by Rac1 inhibitor NSC23766 is that this inhibitor does not target the Rac-specific GEFs involved in cancer cell migration. Further studies are required to elucidate the details of the mechanism of action of this inhibitor. However, our data limit to explain a mechanism on inhibition of cancer cell migration via Rap1 and Tβ4 up-regulation by blocking Rac1. It also has to be defined by considering other signaling pathway.

While questions remain regarding the mechanisms underlying Tβ4 activity, this work provides clear evidence of Rac1 and Rap1 GTPase activation by Tβ4 in tumor metastasis and cancer cell migration. Rap1 is also activated by treatment with a Rac1 inhibitor through a Tβ4-mediated compensatory feedback mechanism in tumor metastasis and cancer cell migration. As Tβ4 proteins form 1:1 complexes with G-actin [27], it is also possible for Tβ4 to play a role in actin reorganization by controlling supply of actin monomer in migration. Together, data demonstrate that Tβ4 could be a novel regulator of crosstalk between Rap1 and Rac1. Simultaneous inhibition of Rap1 and Rac1 may therefore represent an effective therapeutic strategy for inhibiting tumor cell motility. These data suggest that a combination therapy targeting both Rac1 and Rap1 holds potential for the prevention of tumor metastasis and drug resistance *via* compensatory Rap1 activation.

## MATERIALS AND METHODS

### Mice and reagents

Six-week-old male C57BL/6J mice were purchased from Daehan Biolink (Chungju, Korea). Mice were maintained in accordance with the guidelines of the Institutional Animal Care and Use Committee, Sejong University. Tβ4-TALEN for the inhibition of Tβ4 expression was purchased from BMS Co. Ltd. (Seoul, Korea). All mouse experiments were carried out in strict accordance with the guidelines of the Animal and Plant Quarantine Agency, Korea. Relevant study protocols were approved by the Institutional Animal Care and Use Committee, Sejong University (Permit Number: SJ-20120604).

Anti-Rac1 antibodies were obtained from Cell Signaling (Beverly, MA, USA). Additional antibodies against Rac1, Rap1, and HIF1-α were purchased from Santa Cruz Biotechnology (Santa Cruz, CA, USA). NSC23766 trihydrochloride was purchased from Axon Medchem (Groningen, Netherlands), and dissolved in dimethyl sulfoxide (DMSO) with the final concentration not exceeding 0.05%. Except where indicated, all other materials were obtained from the Sigma Chemical Company (St. Louis, MO, USA). RalGDS-RBD-GST and PAK-PBD-GST plasmids were kindly provided by Dr. Johannes L. Bos, Department of Physiological Chemistry, University Medical Center Utrecht UMCU, Netherlands, and Dr. Zhijun Luo, Evans Biomedical Research Center, Boston University School of Medicine, USA, respectively. His-Rac1-V12, His-Rac1-N17, and His-Rap1-N17 plasmid vectors were kindly provided by Dr. Eun-Hye Cho, College of Medicine, Ajou University (Suwon, Korea).

### Yeast two-hybrid screening

The cDNA encoding the 97 amino acids spanning the Rap binding domain of human RalGDS (RBD) [46, 47] was cloned into the pLexA vector in-frame with the LexA DNA-binding domain. HeLa cDNA was ligated into the pJG4–5 vector and fused with the N-terminus of *Escherichia coli* acid blob domain B42. A HeLa cDNA library and bait plasmid pLexA-RBD were then introduced into yeast strain EGY48, which encodes two reporter genes, LEU2 and LacZ, which are regulated by the LexA-B42 protein complex. Transformation was carried out using the lithium acetate method [48]. Leucine-positive colonies were identified by a filter-lifting assay for β-galactosidase activity. Library-derived DNA was prepared from candidate clones, and then subjected to DNA sequencing.

### *In vivo* tumor metastasis

To examine lung metastasis formation, B16F10 mouse melanoma cells were cultivated *in vitro* in log phase. 200,000 B16F10 cells were suspended in 0.2-mL sterile saline and injected into the tail vein of wild-type or Tβ4-Tg C57BL/6 mice. The Rac1 inhibitor NSC23766 was dissolved in saline, and administered intraperitoneally at a dose of 2.5 mg/kg at 12-h intervals for 12 d, beginning on the day of B16F10 injection. The mice were humanely euthanized by cervical dislocation at 14 or 19 d post-injection of B16F10 cells. Lung metastasis was assessed by counting tumor colonies under a dissection microscope [37] and expressed as number of lung metastasis per 100 mm2.

### Cell culture

Human cervical cancer cells, HeLa (ATCC # CCL-2) were obtained from the Korea Research Institute of Bioscience and Biotechnology (KRIBB) cell bank (Daejeon, Korea). B16F10 mouse melanoma cells were obtained from the Korea Institute of Radiological and Medical Science (KIRMS) cell bank (Seoul, Korea). HeLa and B16F10 cells were maintained and cultured in Dulbecco's modified Eagle's medium (DMEM) supplemented with 10% heat-inactivated fetal bovine serum (GIBCO, Grand Island, NY, USA), 2 mM L-glutamine, 100 units/mL penicillin, and 100 units/mL streptomycin (GIBCO). Cells were incubated at 37°C in a humidified incubator, with a 5% CO_2_ atmosphere defined as ‘normoxia.’

The Tβ4 gene was overexpressed or knock-downed by transfection with either pCMV-Tβ4 plasmid or Tβ4-siRNA duplex, respectively [49]. The siRNA sequence was 5′-ccgatatggctgagatcga-3′, which was purchased from Bioneer Corp (Daejeon, Korea).

### Hypoxia treatment

For incubation under hypoxic conditions, cells were placed in an atmosphere of 1% O_2_, 5% CO_2_, 10% H_2_, and 84% N_2_ with intermittent flushing with nitrogen, sealed, and then maintained in a humidified incubator at 37°C in a hypoxia chamber (Forma Anaerobic System, Thermo Electron Corporation, MA, USA). Hypoxia-treated cells were harvested in the hypoxia chamber to prevent the rapid degradation of hypoxia-responsive molecules. For hypoxia-reoxygenation studies, cells were exposed to hypoxia (0.5% O_2_) for an appropriate amount of time in an anaerobic incubator, and then incubated at 37°C under normoxic conditions.

### Reverse transcriptase polymerase chain reaction (RT-PCR)

Total RNA was isolated from HeLa cells using TRIzol reagent (Invitrogen, Carlsbad, CA). cDNA was synthesized from 1 μg total RNA, using oligo-dT_18_ primers and reverse transcriptase in a final volume of 21 μL (Bioneer Corp, Daejeon, Korea). For standard PCR, 1 μL of the first-strand cDNA product was then used as a template for PCR amplification with Taq DNA polymerase (Cosmogenetech Co. Ltd., Seoul, Korea). PCR amplification was performed using oligonucleotides specific for human (h) Tβ4 (forward: 5′-aca aac ccg ata tgg ctg aga tcg aga a-3′, reverse: 5′-ctt gct tct cct gtt caa tc-3′), hGAPDH (forward: 5′-gaa ggt gaa ggt cgg agt c-3′, reverse: 5′-gaa gat ggt gat ggg att tc-3′), mouse (m) Tβ4 (forward: 5′-aca aac ccg ata tgg ctg aga tcg aga a-3′, reverse: 5′-gcc agc ttg ctt ctc ttg tt-3′), and mGAPDH (forward: 5′-tcc acc ctg ttg ctg ta-3′, reverse: 5′-acc aca gtc cat gcc atc ac-3′). PCR products were detected by agarose gel electrophoresis.

### Real-time reverse transcription-PCR analysis

To perform real-time reverse transcription-PCR, total cellular RNA (5 μg) was reverse transcribed into cDNA using SuperScript II (Invitrogen, Carlsbad, CA). Real-time PCR was performed using the CFX96 Touch™ Real-Time PCR Detection System (Bio-Rad laboratories, Hercules, CA, USA). The RT reaction product (100 ng) was amplified with THUNDERBIRD SYBR qPCR Mix (TOYOBO CO., Osaka, Japan) using primers specific for target genes, hTβ4 (forward: 5′-aca aac ccg ata tgg ctg aga tcg aga a-3′, reverse: 5′-ctt gct tct cct gtt caa tc-3′), hGAPDH (forward: 5′-gta tga caa cag cct caa gat-3′, reverse: 5′-agt cct tcc acg ata cca aa-3′), mTβ4 (forward: 5′-aca aac ccg ata tgg ctg aga tcg aga a-3′, reverse: 5′-gcc agc ttg ctt ctc ttg tt-3′), and mGAPDH (forward: 5′-gaa gcc cat cac cat ctt-3′, reverse: 5′-gac tcc acg aca tac tca g-3′). Samples were heated to 95°C for 1 min and amplified for 40 cycles followed by a final extension step of 72°C for 10 min. GAPDH was used as an internal control. Relative quantification of each mRNA was analyzed by the comparative threshold cycle (CT) method.

### Cancer cell migration assay

Cancer cell migration was measured as described previously, with minor modifications [37, 41]. Briefly, when HeLa cells reached confluency in a 35-mm culture dish (Corning, NY, USA), three wound lines in the form of a cross were made by scratching the cellular layer with a plastic pipette tip. Floating cells were then washed out, and fresh medium was added. Cells were then incubated under normoxic condition. Narrowing of the wound was then monitored using a phase-contrast microscope from 6 h after the scratch. The size of the wound at each time point was then quantified using NIH image analysis software (Image J, version 1.62), and compared with that in the initiation of cancer cell migration.

### Measurement of active GTP-bound Rap1 or Rac1

The level of active GTP-bound Rap1 and Rac1 was determined using the GST-pulldown assay, as described previously [42]. Briefly, cells were harvested and lysed. Clarified cell lysates were then incubated with either GST-PBD of PAK (for Rac1) or GST-RBD of RalGDS (or Rap1), in the presence of glutathione immobilized beads (Santa Cruz Biotechnology, CA) at 4°C for 1 h with rotation. Unbound proteins were removed by centrifugation. Samples were then washed with 1X PBS, eluted in 3X SDS sample buffer, and run on an SDS-polyacrylamide gel electrophoresis (SDS-PAGE) gel. Rac1-GTP and Rap1-GTP were detected by western blotting using antibodies specific for Rac1 or Rap1.

### Western blot analysis

Western blots were performed using a standard protocol. HeLa cells were harvested, and then lysed in ice-cold lysis buffer, containing 0.5% Nonidet P-40 (v/v) in 20 mM Tris-HCl (pH 8.3), 150 mM NaCl, protease inhibitors [2 μg/mL aprotinin, pepstatin, 1 μg/mL leupeptin, and 1 mM phenylmethyl sulfonyl fluoride (PMSF)], 1 mM Na_4_VO_3_, and a phosphatase inhibitor. Lysates were incubated for 1 h on ice prior to centrifugation at 13,000 rpm for 10 min at 4°C. Sample protein concentrations were measured using Pierce™ BCA protein assay kit. Proteins in the supernatant were denatured by boiling for 5 min in SDS sample buffer, separated on a 12% SDS-PAGE gel, and then transferred to nitrocellulose membranes by elector-blotting. Following transfer, equal loading was verified by Ponceau S staining. The membranes were then blocked with 5% skim milk in Tris-buffered saline with Tween 20 (TBST; 10 mM Tris-HCl (pH 7.6), 150 mM NaCl, 0.5% Tween 20), and incubated with the indicated antibodies. Bound antibodies were visualized with HRP-conjugated secondary antibodies with the use of enhanced chemiluminescence (ECL) substrate. Immunoreactive bands were detected using X-ray film.

### Statistical analyses

Experimental differences were examined using ANOVA and Students' *t*-tests, as appropriate. *P* values of < 0.05 were considered to indicate significance.

## SUPPLEMENTARY FIGURES


