# Correction: Hypoxia/reoxygenation-experienced cancer cell migration and metastasis are regulated by Rap1- and Rac1-GTPase activation *via* the expression of thymosin beta-4

**DOI:** 10.18632/oncotarget.26543

**Published:** 2018-12-25

**Authors:** Jae-Wook Lee, Yun-Kyoung Ryu, Young-Hoon Ji, Joo Hyun Kang, Eun-Yi Moon

**Affiliations:** ^1^ Department of Bioscience and Biotechnology, Sejong University, Seoul 143–747, Korea; ^2^ Research Center for Radiotherapy, Korea Institute of Radiological and Medical Science, Seoul 139–709, Korea; ^3^ Molecular Imaging Research Center, Korea Institute of Radiological and Medical Science, Seoul 139–709, Korea

**This article has been corrected:** The correct figure 4G is given below. This correction is about data duplication: the original images replaced [originally Figure 4G row 1 image 2&3] are the same images in Figure 4E [row 1 image 2 and row 2 image 2] and the original images replaced [originally Figure 4G row 2 image 2&3] are the same images in Figure 4E [row 2 image 3 and row 1 image 3]. The authors apologize for the oversight. The authors declare that this correction does not affect the description, interpretation, or the original conclusions of the manuscript.

**Figure 4:**
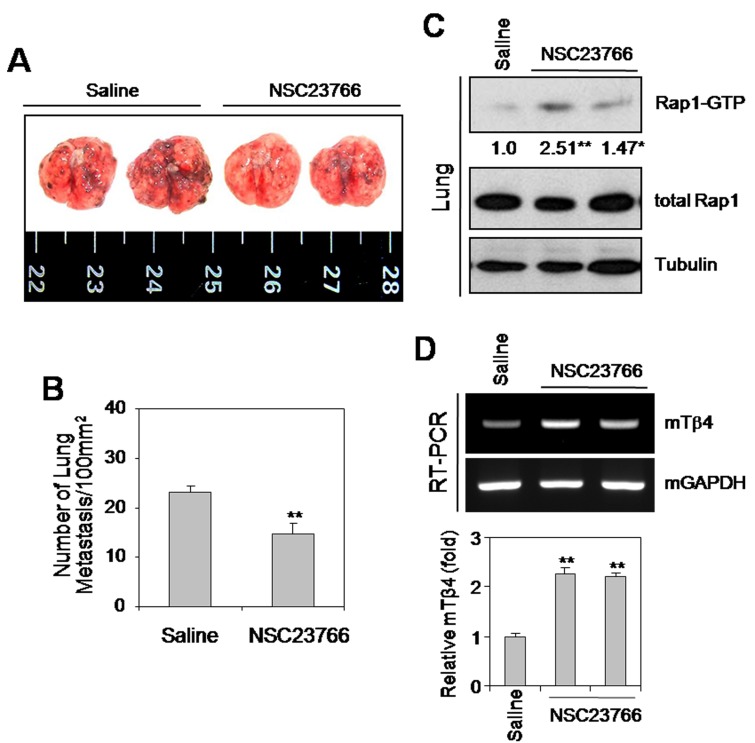

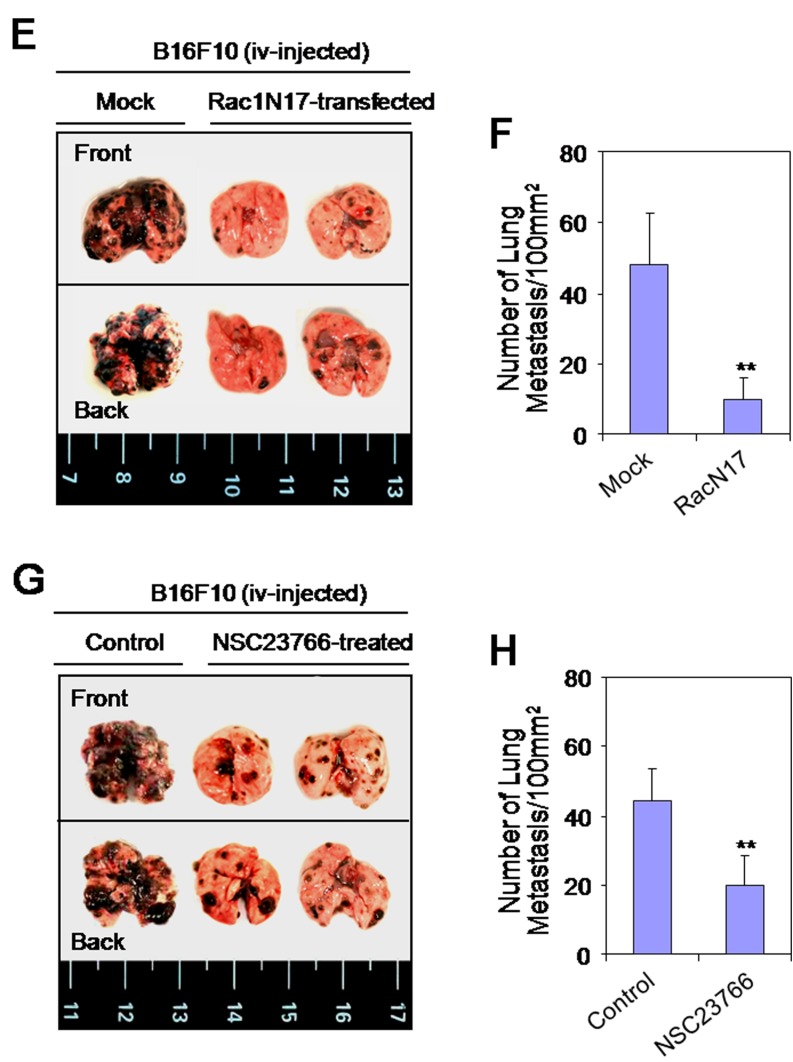
The Rac1-GTPase inhibitor, NSC23766, or Rac1N17 inhibited tumor metastasis *in vivo* **(A–D)** B16F10 cells were cultivated *in vitro* to log phase. Five wild-type C57BL/6 mice were inoculated with 2 × 10^5^ B16F10 cells by tail vein injection. NSC23766 was then injected intraperitoneally at a dose of 2.5 mg/kg every 12 h for 12 d. Mice were then sacrificed on day 19, and examined for lung metastasis (A). The number of lung metastases was assessed by counting tumor colonies under a light dissection microscope (B). Rap1 activity in NSC23766-treated and control B16F10 tumor-bearing mouse lung tissues was detected by GST-pulldown and western blotting. Band intensities were normalized relative to controls using NIH image analysis software (Image J, version 1.62). Fold increases relative to the control are indicated under each band (C). RNA was isolated from control and NSC23766-administered mouse lung tissues. Tβ4 transcript levels were measured by RT-PCR (D, top) or realtime PCR (D, bottom). Data in bar graph are presented as means ± SD (B and D). *p < 0.05; **p < 0.01 relative to saline control (B–D). **(E–H)** B16F10 cells were transfected with Rac1N17 (E and F), or treated with NSC23766 (G and H) for 24 h. Five wild-type C57BL/6 mice were inoculated with 2 × 10^5^ Rac1N17-transfected (E and F) or NSC23766-treated (G and H) B16F10 cells by tail-vein injection. Mice were then sacrificed on day 14, and examined for lung metastasis. (E and G). The number of lung metastases was assessed by counting tumor colonies under a light dissection microscope. Data in bar graph are presented as means ± SD (F and H). **p < 0.01 relative to mock-treated (F) or B16F10 cell-injected controls (H). Data shown are representative of three independent experiments (A-H).

Original article: Oncotarget. 2015; 6:9820-9833. https://doi.org/10.18632/oncotarget.3218

