# Supplementary material for: Hypoxia/reoxygenation-experienced cancer cell migration and metastasis are regulated by Rap1- and Rac1-GTPase activation via the expression of thymosin beta-4
Source: Oncotarget. 2015 Mar 23;6(12):9820–33. doi: 10.18632/oncotarget.3218 (PMC4496400; doi:10.18632/oncotarget.3218)
Supplement: Supplementary file 1 [file oncotarget-06-9820-s001.pdf]

SUPPLEMENTARY FIGURES

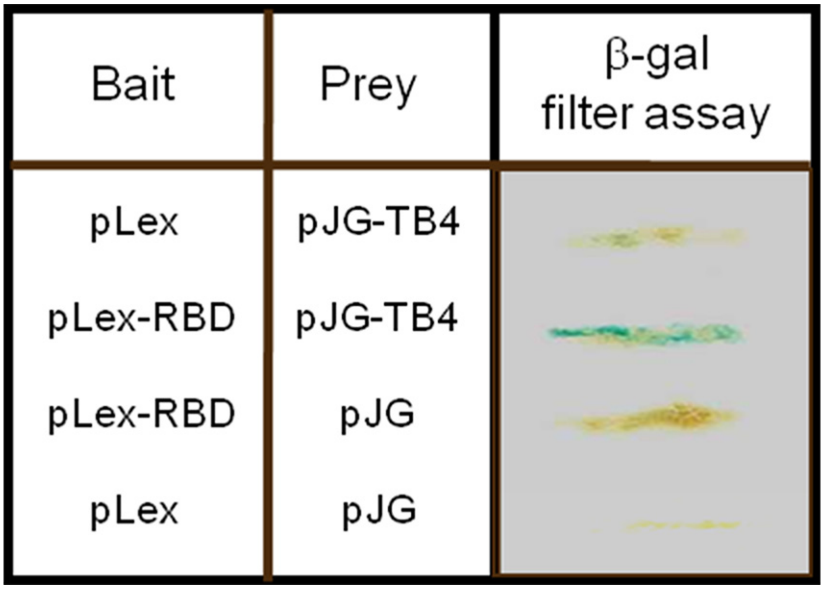

Supplementary Figure 1: Thymosin beta-4 (Tβ4) binds a Ras-binding domain (RBD) in a yeast two-hybrid assay. The binding of Tβ4 to the RBD domain was confirmed by the exchange of vectors. Bait RBD was cloned into the pJG4–5 vector, which was used for cDNA expression, and Tβ4 was transferred into the pLexA vector (prey). The indicated plasmids were transfected into yeast strain EGY48, and plated on SD/gal/raf/-His/-Trp/-Leu/-Ura medium to select for plasmids expressing interactive hybrid proteins (see Materials and Methods). β-gal lift assays were performed; the blue color indicates a positive signal in the assay.

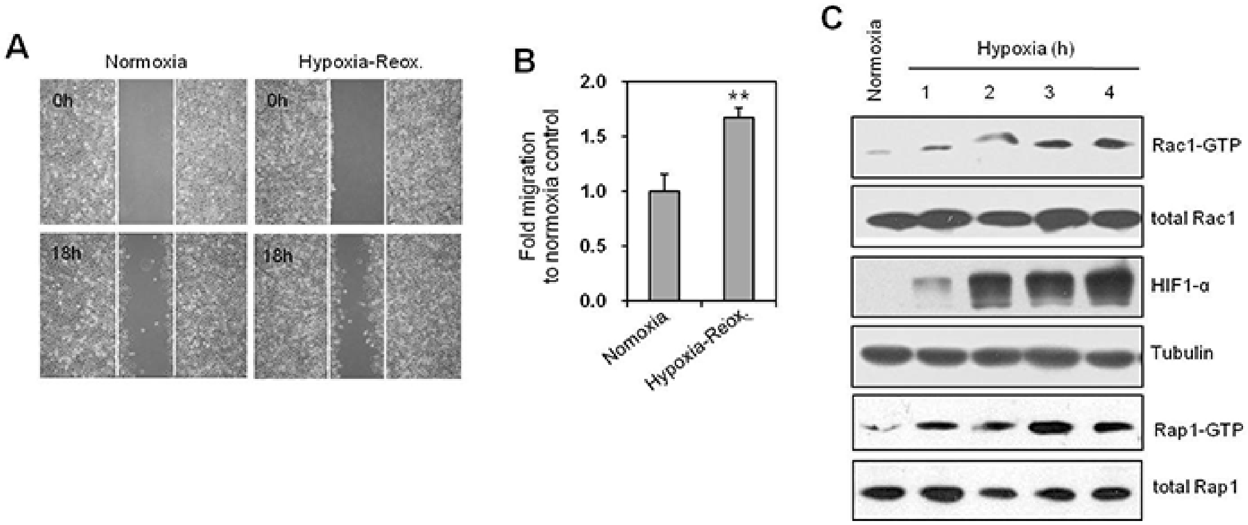

Supplementary Figure 2: Cell migration is increased under hypoxia-reoxygenation conditions *in vitro*. **(A)** HeLa cells were cultured until confluency on 35-mm^2 dishes, and incubated under normoxic conditions. Hypoxia conditioning was carried out by incubation in a 1% O_2 hypoxia chamber for 45 min. A confluent monolayer of HeLa cells was then scratched with a sterile pipet tip and incubated in normoxia for 18 h. The migration of cells into the space left by the scratch was photographed using a phase-contrast microscope at 200× magnification. **(B)** The empty area remaining at each time point was quantified using NIH image analysis software (Image J, version 1.62), and compared to that of the 0-h time point. Data are presented as means ± SD. $^{**}p < 0.01$, relative to controls. **(C)** HeLa cells were incubated under hypoxia for the indicated times (1–4 h), and harvested in a hypoxia chamber. Rac1 and Rap1 activities were measured by GST-pulldown, and assayed for HIF1-α abundance by western blotting.
